# Life Table Parameters and Digestive Enzyme Activity of *Araecerus fasciculatus* (Coleoptera: Anthribidae) Feeding on Different Stored Products

**DOI:** 10.3390/insects16040428

**Published:** 2025-04-18

**Authors:** Lingyan Jian, Yuping Yang, Songhai Xie, Yibin Lou, Ling Chen, Fanglian Dai, Paraskevi Agrafioti, Yu Cao, Christos G. Athanassiou, Can Li

**Affiliations:** 1 Guizhou Provincial Key Laboratory for Rare Animal and Economic Insect of the Mountainous Region, Guizhou Key Laboratory of Agricultural Biosecurity, Guiyang University, Guiyang 550005, China; jly2933727756@163.com (L.J.); 13037865352@163.com (Y.Y.); 13954099591@163.com (S.X.); hmlyb031027@163.com (Y.L.); chenling042457@163.com (L.C.); 18744875950@163.com (F.D.); lican790108@163.com (C.L.); 2 Laboratory of Entomology and Agricultural Zoology, Department of Agriculture, Crop Production and Rural Environment, University of Thessaly, 38446 Nea Ionia, Greece; agrafiot@uth.gr

**Keywords:** coffee bean weevil, stored-product pest, oviposition, population development, digestive physiology, host suitability

## Abstract

*Araecerus fasciculatus* (De Geer, 1775) is an important stored-product pest worldwide. In this study, the development time, survival rate, oviposition, and digestive enzyme (*α*-amylase, cellulase, pepsin, and lipase) activities of *A. fasciculatus* fed on five commodities (coffee, jujube, maize, wheat, and kansui) were investigated. Our results showed that the developmental duration of *A. fasciculatus* from egg to adult was shortest on coffee beans (51.41 days) and longest on kansui (69.65 days). The survival rate of *A. fasciculatus* adults was lowest on kansui (42.22%) and highest on coffee beans (63.33%). Significant differences in fecundity were also observed, with the greatest number on coffee beans (80.78 eggs/female) and the lowest on kansui (50.43 eggs/female). *Araecerus fasciculatus* showed the greatest intrinsic rate of natural increase (*r*_m_) on coffee beans (0.141), followed by jujube (0.129), maize (0.117), wheat (0.105), and kansui (0.097). The net productive rate (*R*_0_) showed a similar trend to *r*_m_, with values of 48.42, 42.53, 35.39, 27.53, and 21.47, respectively, on these stored products. Although no significant differences were observed in the lipase activities when *A. fasciculatus* was fed on different stored products, activities of *α*-amylase, pepsin, and cellulase were highest on coffee beans and lowest on kansui. The variation in the population development of *A. fasciculatus* associated with different foods may be related to its digestive enzyme performance. These results indicated that coffee beans were the most suitable host food, while kansui was the least suitable for the development of *A. fasciculatus*.

## Simple Summary

*Araecerus fasciculatus* (De Geer, 1775) is a globally significant pest of stored products. Dietary differences can influence the physiological and ecological traits of insects. In this study, we examined the population development and digestive enzyme activities of *A. fasciculatus* reared on five distinct commodities: coffee, jujube, maize, wheat, and kansui. The results revealed that *A. fasciculatus* fed on coffee beans exhibited the shortest developmental period, highest adult survival rate, and highest fecundity. Conversely, kansui supported the longest developmental period, the lowest survival rate, and the lowest fecundity. Similarly, the highest values for both the intrinsic rate of natural increase (*r_m_*) and the net reproductive rate (*R*_0_) were recorded on coffee beans, whereas the lowest values were observed on kansui. Furthermore, when fed on different stored products, *A. fasciculatus* demonstrated the highest activities of key digestive enzymes, including α-amylase, pepsin, and cellulase, on coffee beans, while the lowest enzyme activities were detected on kansui. These findings indicate that dietary variations significantly influenced the metabolism of *A. fasciculatus*, which in turn may profoundly affect its population development. In summary, the results suggest that coffee beans represent the most suitable host food for the development of *A. fasciculatus*, whereas kansui is the least suitable.

## 1. Introduction

The coffee bean weevil, *Araecerus fasciculatus* (De Geer, 1775), is an economically important, cosmopolitan stored-product pest with a worldwide distribution, especially in subtropical and tropical areas. This species also has a wide range of food preferences, and attacks more than 100 types of stored products, such as dry cassava, cocoa, nutmeg, coffee, sweet potatoes, areca nut, maize, and traditional Chinese medicine materials [1,2,3]. *Araecerus fasciculatus* can cause considerable damage to stored products of plant origin; for example, this pest has been shown to damage 20.60–91.51% of stored cassava [2]. Therefore, the damage caused by this pest can result in serious quantitative losses and qualitative degradations in a wide range of stored products [1,4].

Fumigation with phosphine has been the most important method for controlling the vast majority of stored-product pests, including *A. fasciculatus* [1,5]. However, the extensive use and continuous and/or unsuccessful application of common fumigants, e.g., methyl bromide and phosphine, have resulted in serious problems, including damage to the ozone layer, pest resistance, lethal effects on non-target organisms, and residues that are harmful to humans and the environment [6,7,8,9]. Hence, the development of alternative approaches is needed to improve the control of insect pests in the stored-product community; however, in the case of *A. fasciculatus*, such efforts have been hindered because not enough is known about its key biological parameters.

Previous studies on different types of stored products have shown that these commodities can notably affect the demographic parameters of certain stored-product insect pest species [10,11,12,13]. Nevertheless, considering that there are few data available in the case of *A. fasciculatus* on this topic, here, we investigated the development, survival, fecundity, and other life table characteristics of this species on five selected stored products (coffee beans, jujube, maize, wheat, and kansui). Owing to the important role that digestive enzymes play in digestion and nutrient absorption after food intake, they have been employed as regulatory factors to govern the extent to which nutrients occurring in surpluses or deficits are eaten by insects fed on different food sources [14,15] and were found to directly affect their population development [16,17,18,19,20]. Therefore, in this study, we also tested the differences in the activity of digestive enzymes [*α*-amylase (*α*-AMS), cellulase (CL), pepsin (PEP), and lipase (LPS)] in *A. fasciculatus* fed on the five stored products listed above. Our results will assist in the prediction of *A. fasciculatus* occurrences on different stored products and contribute to a better understanding of the host adaptation of *A. fasciculatus* and related adaptive mechanisms in the context of physiological ecology. These results are expected to provide an enhanced understanding of the varying degrees of damage caused by *A. fasciculatus* to various kinds of commodities, illustrating its food preferences.

## 2. Materials and Methods

### 2.1. Insect Rearing

*Araecerus fasciculatus* was reared in 5 L glass jars covered with muslin cloth to prevent insect escape and ensure ventilation as per our previous study [21]. Insects were reared at 28 ± 1 °C, a 75 ± 5% relative humidity (RH), and under a 16:8 h (light/dark) photoperiod in a climate chamber. *A. fasciculatus* has been in culture in our laboratory since 2022 and is routinely maintained on hulled Daohuaxiang Rice (Wuyoudao No. 4), with a moisture content of 12–14%.

### 2.2. Stored Products

Five different stored products, namely coffee beans (*Coffea liberica* Bull ex Hiern), jujube (*Ziziphus jujuba* Mill. var. Jinsi No. 4), maize (*Zea mays* L. var. Jinfuyu No. 66), wheat (*Triticum aestivum* L. var. Hualiang No. 517), and kansui (*Euphorbia kansui* S. L. Liou ex S. B. Ho), with moisture contents of 10–12%, 20–25%, 13–14%, 12–13%, and 11–15%, respectively, were purchased from the Guiyang Grain Commodity Market (Guiyang City, Guizhou Province, China). All commodities were pesticide- and pest-free.

### 2.3. Development and Survival of Araecerus fasciculatus

Groups of *A. fasciculatus* adults (200 individuals, male/female = 1:1) were placed into glass jars (2.5 L) for oviposition, and each jar contained 10.0 g of one of the five selected stored products. *A. fasciculatus* adults were allowed to oviposit on these stored products for 48 h and were then removed. Newly laid eggs were collected from each stored product for further investigation. A total of 60 eggs originating from different cultures were transferred carefully into Petri dishes (85 mm in diameter and 15 mm in height) containing 10.0 g of each stored material, as suggested in previous studies [13,20]. The development and survival of each immature stage (from egg to adult) of *A. fasciculatus* were checked and recorded daily. Three replicates were conducted for each stored material.

### 2.4. Fecundity

At emergence, *A. fasciculatus* adults were paired (1 female/1 male) to assess their fecundity (total number of eggs laid) on each of the five stored products listed above. Each pair of *A. fasciculatus* was transferred into a single Petri dish, as mentioned above, containing 15.0 g of uninfested stored materials for oviposition [13]. Each pair of adults was transferred daily to a new Petri dish containing correspondingly fresh stored product, and all rearing materials were checked daily to count the number of eggs. The fecundity of each female was recorded until death. The offspring of *A. fasciculatus* from each stored product were also reared to adulthood to identify their sex and then calculate their offspring sex ratio. These fecundity assays were conducted for one replicate of 20 pairs of beetles, totaling 60 pairs per product for the three replicates on each stored product.

### 2.5. Life Table Parameters

On the basis of the survivorship and reproductive data, life tables for *A. fasciculatus* were constructed according to the methods of Ren et al. and Naseri and Majd-Marani [13,20]. Life table parameters were calculated as follows:

(1) $$R_{0}=\sum l_{x}m_{x}$$

(2) $$r_{m}=\frac{\ln R_{0}}{T}$$

(3) $$\lambda =e^{r_{m}}$$

(4) $$T=\frac{\sum l_{x}m_{x}x}{\sum l_{x}m_{x}}$$

(5) $$\mathrm{DT}=\frac{\ln2}{r_{m}}$$

where *x* represents the time interval in units per day; *l_x_* represents the survival rate of any one individual during the time *x*; and *m_x_* represents the average number of female offspring produced by *A. fasciculatus* on each stored product.

### 2.6. Activity Assays of Digestive Enzymes

#### 2.6.1. Enzyme Extract Preparation

Enzyme extracts were prepared from *A. fasciculatus* adults fed on each of the five stored products. Protein extraction protocols were performed using a total protein quantitative assay (Nanjing Jiancheng Bioengineering Institute, Nanjing, China) [22]. Protein concentrations were determined according to the Bradford method using bovine serum albumin as the standard [23]. If the enzyme activity was not measured immediately, the extracts were stored at −20 °C until used.

#### 2.6.2. Digestive Enzyme Activity Assay

Procedures for the activity assays of each enzyme (*α*-AMS, LPS, PEP, and CL) were performed according to the manufacturer’s instructions for the relevant kits (Nanjing Jiancheng Bioengineering Institute, Nanjing, China). The optical density was recorded using a microplate reader (SpectraMax M2, Molecular Devices, San Jose, CA, USA). There were three repetitions for the activity assays of each digestive enzyme.

## 3. Statistical Analyses

Data were analyzed using SPSS software (version 19.0; SPSS, Chicago, IL, USA), and data were checked for normality and homoscedasticity before being analyzed. One-way analyses of variance (ANOVAs) followed by Tukey’s honestly significant difference (HSD) test were used to determine significant differences in development, survival, oviposition, and other life table parameters, as well as digestive enzyme activities, in *A. fasciculatus* among different stored products (*p* < 0.05).

## 4. Results

### 4.1. Development

The developmental periods of eggs (*F*_4,10_ = 43.48, *p* < 0.01), larvae (*F*_4,10_ = 285.21, *p* < 0.01), and pupae (*F*_4,10_ = 36.60, *p* < 0.01) of *A. fasciculatus* differed significantly among the five stored products (Table 1). There were also significant differences in the duration of development from egg to adult (*F*_4,10_ = 736.35, *p* < 0.01) in *A. fasciculatus* among these stored products. The developmental times from egg to adult were 51.41 days on coffee beans, 56.16 days on jujube, 59.67 days on maize, 64.25 days on wheat, and 69.65 days on kansui.

### 4.2. Survival

**Figure 1:**
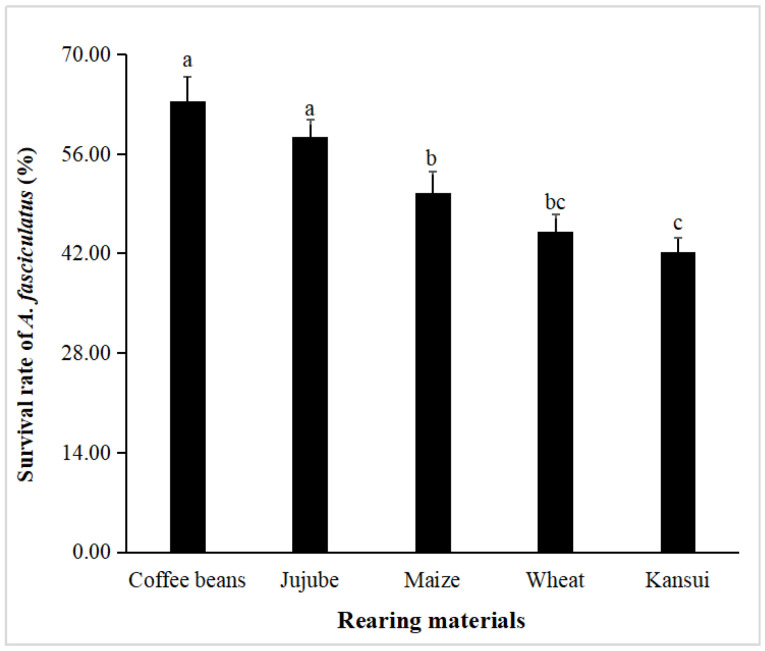
Survival rate (%) of immature stages of *Araecerus fasciculatus* raised on different stored products. Data are presented as mean ± standard error. Different letters above bars indicate significant differences among values (Tukey’s test, *p* < 0.05).

There were significant differences in the survival rate of the immature stage (egg to adult) (*F*_4,10_ = 31.15, *p* < 0.01) of *A. fasciculatus* among the five selected stored products (Figure 1). *A. fasciculatus* adults showed the highest survival rate (63.33%) on coffee beans, followed by 58.33% on jujube, 50.55% on maize, 45.00% on wheat, and 42.22% on kansui.

### 4.3. Oviposition and Adult Longevity

The fecundity of *A. fasciculatus* differed significantly among the tested stored products (*F*_4,10_ = 119.94, *p* < 0.001) (Table 2). Tested stored products were ranked, from the highest number of *A. fasciculatus* eggs per female to the lowest, as follows: coffee beans (80.78), jujube (75.62), maize (68.04), wheat (60.46), and kansui (50.43). Male *A. fasciculatus* had the highest longevity on coffee beans (36.56 d), which was not significantly different from that on jujube (35.57 d), maize (35.17 d), or wheat (34.50 d) (*F*_4,10_ = 7.83, *p* = 0.004). The lowest longevity of male *A. fasciculatus* was on kansui (32.80 d). Similar results were also obtained for the longevity of female *A. fasciculatus* among these stored products (*F*_4,10_ = 26.66, *p* < 0.001).

### 4.4. Life Table Parameters

There were significant differences in the net productive rate (*R*_0_) and the intrinsic rate of natural increase (*r*_m_) among coffee beans, jujube, maize, wheat, and kansui, with *R*_0_ values of 48.42, 42.53, 35.39, 27.53, and 21.47 (*F*_4,10_ = 492.76, *p* < 0.001), respectively, and *r*_m_ values of 0.141, 0.129, 0.117, 0.105, and 0.097 (*F*_4,10_ = 157.15, *p* < 0.001), respectively (Table 3). Additionally, the finite rate of increase (*λ*) for *A. fasciculatus* differed significantly (*F*_4,10_ = 35.27, *p* < 0.001) among these five stored products. We also observed significant differences in the mean generation time (*T*) (*F*_4,10_ = 25.59, *p* < 0.001) of *A. fasciculatus* among the stored products, although the *T* values had the opposite trend to those of *λ*, *R*_0_, and *r*_m_.

### 4.5. Digestive Enzyme Activity Bioassays

**Figure 2:**
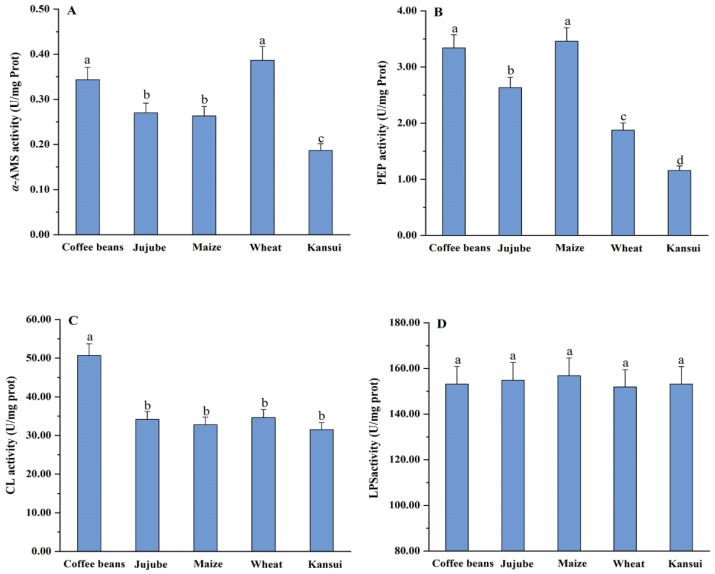
Digestive enzyme activities in *Araecerus fasciculatus* adults on different stored products. Data are presented as mean ± standard error. Different lowercase letters above bars indicate significant differences (one-way ANOVA followed by Tukey’s HSD test, *p* < 0.05). *α*-AMS = *α*-amylase, PEP = pepsin, CL = cellulase, and LPS = lipase.

The *α*-AMS activities of *A. fasciculatus* adults differed significantly among the five stored materials (*F*_4,10_ = 32.90, *p* < 0.001) (Figure 2A). The highest *α*-AMS activity was on wheat (0.39 U/mg protein), which was not significantly different from that on coffee beans, but was 1.44, 1.50, and 2.05 times greater than that on jujube, maize, and kansui, respectively. The highest PEP activity of *A. fasciculatus* was on maize (3.46 U/mg protein), which was also not significantly different from that on coffee beans (3.34 U/mg protein), but was 1.32, 1.85, and 2.98 times greater than that on jujube, wheat, and kansui (*F*_4,10_ = 288.05, *p* < 0.001), respectively (Figure 2B). There was a significant difference in the CL activity when *A. fasciculatus* was fed on the different stored products (*F*_4,10_ = 95.93, *p* < 0.001), with the highest CL activity at 50.65 U/mg protein on coffee beans (Figure 2C). We observed no significant differences in CL activity in *A. fasciculatus* fed on the remaining four stored materials. Finally, no significant difference was observed in the LPS activities of *A. fasciculatus* among the five stored materials (*F*_4,10_ = 1.17, *p* = 0.39) (Figure 2D).

## 5. Discussion

A series of studies have indicated that different food sources can influence the development, survival, and reproductive characteristics of stored-product insect pests [10,16,24,25]. In this study, we observed significant differences in the developmental periods, survival rates, and fecundity of *A. fasciculatus* among the five different products tested, indicating the specific food suitability of this species and its potential to be an important cause of infestation for specific commodities. Therefore, according to the life history parameters of *A. fasciculatus*, our results indicated that coffee beans were the most suitable diet for the population development of this pest species, while kansui was the least suitable one. Considering that coffee is a high-value commodity of global importance, and at the same time is a “niche” commodity for most stored-product insects [26], the fact that *A. fasciculatus* can develop so easily in coffee should be carefully considered.

Thus far, little information has been reported about the influence of different stored materials on the population development of *A. fasciculatus*. Chijindu and Boateng reported that there were significant differences in the development periods (from egg to adult) of *A. fasciculatus*, ranging from 55.5 to 61.7 days [4], on different processed chips, and temperature could significantly affect the hatching rate and period of *A. fasciculatus* eggs [27]. Humidity also significantly influenced the development, survival, and oviposition of *A. fasciculatus*, with *r*_m_ ranging from 0.197 to 0.322 and *R*_0_ from 9.653 to 73.493 under different humidities (30–90%) at 27 °C [28]. We obtained similar results for the population development of *A. fasciculatus* in the different stored products in our study, with the population development and sizes (from highest to lowest) of *A. fasciculatus* showing the trend of coffee beans > jujube > maize > wheat > kansui, according to the *R*_0_ (ranging from 21.47 to 48.42) and *r*_m_ (ranging from 0.097 to 0.141) values, which is clearly indicative of the food preferences of this species in terms of both longevity and fecundity.

Previous studies have reported that differences in the seed hardness, macronutrient contents, protein inhibitors, and other physicochemical properties among different stored products affect the development, survival, fecundity, and other parameters of the population development of stored-product insect pests [11,17,20]. Here, because the five stored products investigated in this study were clustered into different families and orders, the differences in their physicochemical characteristics should be further studied to assess their influence on the population development of *A. fasciculatus*. In particular, as the nutrient composition of a food source increases, the growth cycle shortens, and the population size of the insects increases [29,30]. Therefore, from the point of view of nourishment, our results indicate that coffee beans are a better food source, compared with the other four stored materials, to benefit the population development of *A. fasciculatus*. Nevertheless, the initial abiotic characteristics of these commodities, e.g., their initial moisture content, could be partially responsible for the variations reported here.

Food quality can significantly affect the population performance of phytophagous insects, and digestive enzymes contribute to nutrient metabolism in insects after food intake, playing crucial roles in food consumption, absorption, and nutrient utilization [14,15,31]. Here, the *α*-AMS activity level (from highest to lowest) of *A. fasciculatus* on the selected stored materials was ranked in the order of wheat ≥ coffee beans > jujube ≈ maize > kansui, the PEP activity level was maize ≥ coffee beans > jujube > wheat > kansui, and the CL activity level was coffee beans > jujube ≈ maize ≈ wheat ≈ kansui. Related inhibitors in less suitable host plants [32,33,34] may have a detrimental effect on the activities of the digestive enzymes in *A. fasciculatus*. Here, we found that *A. fasciculatus* showed significantly higher activities of digestive enzymes (*α*-AMS, PEP, and CL) in the more suitable host materials (coffee beans). Similar results were also obtained for the lesser grain borer, *Rhyzopertha dominica* (Fabricius) (Coleoptera: Bostrichidae) fed on different rice or barley cultivars [18,19]. The variation in enzyme activities of these stored-product pests may be related to the differences in nutritional components among different stored substances [17,20,31]. However, in this study, the performance of the three digestive enzymes was not in accordance with the degree of the population development of *A. fasciculatus* on the five stored products (coffee beans > jujube > maize > wheat > kansui). Therefore, how these different digestive enzymes may interact to aid in nutrient supply for the population development of *A. fasciculatus* should be further studied, as well as the identification of regulatory genes for these physiological enzymes in *A. fasciculatus* to explore the related molecular mechanisms [35,36].

In summary, our results showed that there was a significant difference in the population development of *A. fasciculatus* among the five different stored products, with coffee beans and kansui revealed to be the most and least susceptible commodities, respectively. Our study highlights the importance of this species at the post-harvest stages of coffee, a product that is not much affected by most stored-product pests. Moreover, we have detected that *A. fasciculatus* can easily develop high population densities in this specific commodity in a relatively short period of time, with devastating consequences. Additionally, our work demonstrates that other commodities, for which there were no data available before now, are also susceptible to this species and may be prone to serious infestations by *A. fasciculatus*.

## Figures and Tables

**Table 1:** Developmental time of *Araecerus fasciculatus* on different stored products.

Diet	Egg Incubation	Larval Period	Pupal Period	Immature Period
Coffee beans	6.12 ± 0.10 d	39.24 ± 0.48 e	6.77 ± 0.12 d	51.41 ± 0.33 e
Jujube	6.48 ± 0.07 c	43.36 ± 0.36 d	7.05 ± 0.04 cd	56.16 ± 0.24 d
Maize	6.82 ± 0.08 b	45.93 ± 0.30 c	7.26 ± 0.04 bc	59.67 ± 0.30 c
Wheat	7.13 ± 0.05 ab	49.82 ± 0.38 b	7.45 ± 0.03 b	64.25 ± 0.17 b
Kansui	7.23 ± 0.08 a	55.12 ± 0.23 a	7.76 ± 0.04 a	69.65 ± 0.24 a

Data are presented as mean ± standard error. Different lowercase letters in the same column indicate significant differences (one-way ANOVA followed by Tukey’s HSD test, *p* < 0.05).

**Table 2:** Fecundity and longevity of *Araecerus fasciculatus* on different stored products.

Diet	Fecundity (Eggs per Female)	Male Longevity (d)	Female Longevity (d)
Coffee beans	80.78 ± 1.47 a	36.56 ± 0.40 a	37.49 ± 0.40 a
Jujube	75.62 ± 0.70 b	35.57 ± 0.52 a	36.15 ± 0.35 ab
Maize	68.04 ± 0.90 c	35.17 ± 0.16 a	35.74 ± 0.33 ab
Wheat	60.46 ± 0.90 d	34.50 ± 0.45 ab	34.87 ± 0.43 b
Kansui	50.43 ± 1.19 e	32.80 ± 0.77 b	32.20 ± 0.39 c

Data are presented as mean ± standard error. Different lowercase letters in the same column indicate significant differences (one-way ANOVA followed by Tukey’s HSD test, *p* < 0.05).

**Table 3:** Life table parameters of *Araecerus fasciculatus* on different stored products.

Diet	*R*_0_	*r*_m_	*λ*	*T*
Coffee bean	48.42 ± 0.48 a	0.141 ± 0.001 a	1.152 ± 0.001 a	27.51 ± 0.32 c
Jujube	42.53 ± 0.54 b	0.129 ± 0.002 b	1.137 ± 0.002 b	29.12 ± 0.44 bc
Maize	35.39 ± 0.52 c	0.117 ± 0.002 c	1.125 ± 0.002 c	30.39 ± 0.37 ab
Wheat	27.53 ± 0.32 d	0.105 ± 0.001 d	1.111 ± 0.001 cd	31.48 ± 0.31 a
Kansui	21.47 ± 0.56 e	0.097 ± 0.001 e	1.101 ± 0.001 d	31.86 ± 0.31 a

Data are presented as mean ± standard error. Different lowercase letters in the same column indicate significant differences (one-way ANOVA followed by Tukey’s HSD test, *p* < 0.05). *R*_0_ = net productive rate, *r*_m_ = intrinsic rate of natural increase, *λ* = finite rate of increase, and *T* = mean generation time.

## Data Availability

The original contributions presented in this study are included in the article. Further inquiries can be directed to the corresponding author.
